# Plasmonic Nanoparticles Driven Enhanced Light Amplification in a Local 2D and 3D Self-Assembly

**DOI:** 10.3390/nano8121051

**Published:** 2018-12-14

**Authors:** Konrad Cyprych, Denis Chateau, Anthony Désert, Stephane Parola, Jaroslaw Mysliwiec

**Affiliations:** 1 Advanced Materials Engineering and Modelling Group, Wroclaw University of Science and Technology, Wyb. Wyspianskiego 27, 50-370 Wroclaw, Poland; konrad.cyprych@pwr.edu.pl; 2 Laboratoire de Chimie, Université de Lyon, Université Lyon 1, CNRS UMR 5182, Ecole Normale Supérieure de Lyon, 46 allée d’Italie, F69364 Lyon, France; denis.chateau@ens-lyon.fr (D.C.); anthony.desert@ens-lyon.fr (A.D.); stephane.parola@ens-lyon.fr (S.P.)

**Keywords:** gold nanoparticles, light amplification, Rhodamine 6G

## Abstract

We present fluorescence and a random lasing enhancement effect due to the interaction between gold nanoparticles (AuNPs) and Rhodamine 6G (Rh6G) dye. Non-covalently bounded dyes in the proximity of nanoparticles are studied in three systems of varying dimensionality: from (i) three-dimensional freely distributed suspensions, through (ii) quasi-two-dimensional multilamellar liposomes, to (iii) solid two-dimensional thin layers. Liposomes facilitate the formation of stable AuNPs/Rh6G composition showing enhanced fluorescence, while solid thin films exhibit plasmon-assisted random lasing.

## 1. Introduction

Utilization of metal nanoparticles (NPs) in the enhancement of fluorescence emission was widely described in literature; however, the main emphasis was often put to the organic or inorganic modified compounds interacting with metal nanoparticles [1,2,3,4,5,6,7,8,9,10,11,12,13,14,15,16,17,18]. The hybrid systems are often devoted to imaging with assistance of gold nanoparticles [19], cancer treatments [20], where NPs introduced to the cancer tissue are responsible for thermolysis of tumor by selective laser irradiation [21,22], sensing or photovoltaics [7,23].

Metal nanoparticles can be introduced in hybrid materials for optical applications in very different ways [18]. Association between a polymer and dispersed metal nanoparticles increase efficiency of solar panels, as it appears in silver and gold NPs in coumarin doped poly(methyl methacrylate) (PMMA). Such modification resulted in appearance of metal enhanced fluorescence (MEF) properties [24]. Nanocomposites are used for sol-gel SiO${}_{2}$ colloid systems. Dye doping form stable material [25], for nonlinear light–matter interactions studies [26,27,28]. Another way to acquire stable hybrid systems showing fluorescence enhancement is the aggregation induced process via surface physical properties [29,30].

In the system exhibiting population inversion of states, it is possible to achieve light amplification, namely amplified spontaneous emission (ASE) [30]. Metal nanoparticles applied for such a purpose induce additional light enhancement utilizing plasmon surface resonance properties [31]. Concomitantly, light scattering also have an additional positive effect on the light amplification, where nanoparticles are acting as centers of constructive scattering responsible for generation of a random lasing phenomenon. This was shown in numerous systems [32,33], for instance in the presence of gold nano-stars [34], TiO${}_{2}$ nanoparticles [35] or dye-doped cellulose [36].

This article focuses on the light amplification assisted by plasmonic gold nanoparticles inserted in composite hybrid materials. The nanoparticles impact on the light amplification, as well as dye-nanoparticle interactions were investigated three systems of varying dimensionality: (a) freely suspended compounds in water at appropriate ratio acting as dispersed 3D system, (b) multilamellar vesicles—a self-confined system of quasi-2D geometry with surface dimensionalities highly exceeding the thickness [37] and (c) polymeric thin film doped with dye and nanoparticles.

## 2. Materials and Methods

Rhodamine 6G (Rh6G), dichloromethane (DCM), α-phosphatidylcholine lipids (α-PC), poly-vinyl alcohol (PVA), tetrahydrofuran (THF, 99%), cetyltrimethylammonium chloride (CTAC) 25% in water, 8-hydroxyquinoline (HQL, 99%), NaBH${}_{4}$ (99.999%) and NaOH (98%) were purchased from Sigma-Aldrich (St. Louis, MO, USA) and used as received. HAuCl${}_{4}$, 3H${}_{2}$O (99.9%) was purchased from Alfa Aesar (Hafel, MA, USA). All materials and suspensions were prepared using Milli-Q water (Merck KGaA, Darmstadt, Germany).

### 2.1. Spherical Gold Nanoparticles (AuNPs) Synthesis

The spherical gold nanoparticles were prepared by a two-step seeds-mediated method, with the advantage of giving highly monodispersed nanoparticles. The seeds were prepared by quickly injecting, under vigorous magnetic stirring, 400 μL of a freshly prepared mixture of NaBH${}_{4}$ 50 mM/NaOH 50 mM into 32 mL of CTAC (66 mM), 320 μL of HAuCl${}_{4}$ (25 mM) and 296 μL of HNO${}_{3}$ (0.25 M). The stirring was stopped after 1 min and the obtained solution was aged at 80 ${}^{\circ }$C for 50 min. The growth solution was prepared by adding 600 μL of concentrated CTAC (0.78 M) and 200 μL of HAuCl${}_{4}$ (25 mM) into 19.4 mL of Milli-Q purified water. Then, the mixture was stirred for 15 min at 60 ${}^{\circ }$C before adding 150 μL of HQL (0.4 M in THF). Finally, spherical gold nanoparticles were synthesized by adding 100 μL of the seeds solution to the growth solution. The nanoparticles were obtained in a form of water suspension stabilized with 23 mM CTAC.

The gold nanoparticles’ synthesis produced monodisperse AuNPs of hydrodynamic diameter equal to d=52.5 nm with polydispersity index of PDI=0.19 evaluated with dynamic light scattering technique (DLS) on Zetasizer Nano Z (Malvern Instruments, Malvern, UK). These AuNPs exhibited a plasmon band centered at $\lambda_{AuNP}=532$ nm with estimated molar absorption coefficient equal to $\varepsilon_{AuNP}=1.72\times 10^{10}$ m${}^{2}$/mol [38].

### 2.2. Dye and Nanoparticles Water Suspension—3D System

Rh6G dyes and AuNPs’ optical properties were tested in a form of water suspension in a concentration of $c_{Rh6G}=10^{-3}$ mg/mL with different AuNP amount ratios in a range $4.8\times 10^{-6}$ to $4.8\times 10^{-11}$ nanoparticles per Rh6G molecule (CTAC concentration increase linearly with AuNPs).

### 2.3. Dye/AuNPs Multilamelar Vesicles—Quasi-2D System

**Figure 1:**
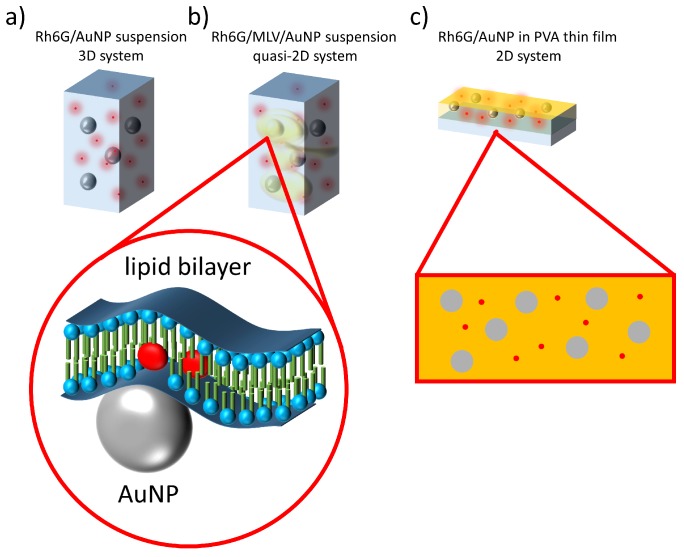
Schematics of experimental systems: (**a**) AuNPs and Rh6G suspended in water; (**b**) AuNPs and Rh6G in MLVs forming quasi-2D system of lipid bilayers; (**c**) PVA solid thin layers with embedded AuNPs and Rh6G.

Multilamellar vesicles (MLVs) were produced using standard preparation protocol for MLVs from α-PC dissolved in DCM [39]. The solvent evaporation formed a lipid thin film casted on the inner glass vial surface [40,41]. The vial containing lipid film was soaked with previously preparing the Rh6G/AuNP water suspension in the appropriate ratio. Vigorous shaking led to formation of MLVs suspended in the water together with AuNPs and Rhodamine 6G. Liposomes’ preparation protocol was intentionally not followed by liposomes’ size calibration. We have prepared samples with constant α-PC lipids concentration of $c_{PC}=1$ mg/mL and Rh6G concentration of $c_{Rh6G}=10^{-3}$ mg/mL, but with different AuNP amount ratios ($4.8\times 10^{-6}$ to $4.8\times 10^{-11}$ nanoparticles per Rh6G molecule). The quality of formed structures was measured with DLS according to the NP free reference samples. Samples containing MLVs with AuNPs and dye were tested in a form of suspension (cf. Figure 1b).

### 2.4. Dye/NPs Polymeric Thin Films—2D System

The further dimension reduction according to the previously described 3D suspension and quasi-2D lipid bilayer systems was done by the formation of AuNPs/Rh6G doped polymeric layers. PVA was used as a host polymer matrix. Mixtures of PVA, Rh6G and AuNPs were prepared at constant weight ratio of dye 2.0%(w/w) to PVA dry mass and PVA equal to 2.0%(w/w) according to water. Different amounts of AuNPs in the range of $4.0\times 10^{4}$–$4.0\times 10^{11}$ particles per unit volume were applied to the mixtures. Water suspensions of mixed compounds were deposited on glass slides by a drop-casting method. The sample deposition was followed by water evaporation in an air atmosphere forming solid thin layers composed of PVA, AuNPs and Rh6G as schematically presented in Figure 1c.

### 2.5. Optical Experiments

The fluorescence properties of the samples were evaluated on the Fluoromax-4 spectrofluorometer (Horiba, Kyoto, Japan) with excitation at 532 nm corresponding to the Rh6G maximum excitation, and plasmon band central wavelength. Light amplification studies in dye-doped PVA thin films were measured in an experimental setup with Nd:YAG pulsed nanosecond laser Surelite II, τ=7 ns (Coherent, Santa Clara, CA, USA) followed by an Optical Parametric Oscillator (Horizon, Continuum, Santa Clara, CA, USA). The optical system for the samples’ excitation was built from a half-wave plate coupled with polarizer responsible for the pumping energy density and polarization state control. Pumping beam was enlarged with an expander followed by a cylindrical lens to form stripe-like beam geometry. By default, the size of excited geometry was equal to 0.5 mm × 2.0 mm and located at the edge of a glass slide (2D systems’ measurements). The emission coming from the sample was collected with an optical fiber spectrometer (Shamrock SR-163, Andor Technology, Belfast, UK) of Δλ=0.1 nm spectral resolution. For the random lasing threshold determination, series of measurements were done in a function of modulated excitation energy density.

The transport mean-free path of light in materials was measured using coherent backscattering experimental technique (CBS) involving intensity measurements at a low backscatter angle of *cw* He-Ne laser emitting at wavelength 632.8 nm, which was out of the dye and nanoparticles’ absorption bands. Samples were placed at a distance *L* = 100 cm from the photomultiplier mounted on the moving stage. Scattering cones were measured for the angle ranging of ω=±15 mrad. The light transport mean-free path $l_{t}$ was calculated according to the analytical equation [42,43]:

(1) $$l_{t}=\frac{0.7\times \lambda }{2\pi \times \Delta \omega },$$

where λ=632.8 nm and Δω is full width at a half maximum of the measured scattering cone.

## 3. Results and Discussion

The three types of sample series have been compared according to the light amplification possibility. There have been tested Rh6G/AuNP composition suspended in water, the same composition in the presence of α-PC MLV, and the system that used PVA as a solid host for the Rh6G/AuNPs. Measured fluorescence properties of all three systems have shown significant differences.

### 3.1. Water Suspensions: 3D and Quasi 2D Systems

The 3D system of Rh6G/AuNPs water suspension has shown a suppressive effect of the nanoparticles to the fluorescence emission, caused by the fluorescence excitation at the plasmon resonance peak wavelength $\lambda_{ex}=532$ nm. Moreover, intensity decrease was the effect of absorption of AuNPs at excitation wavelength. The lowest emission intensity, 75% according to the reference Rh6G fluorescence signal in water, was reached for an AuNP concentration of $4.8\times 10^{-6}$ nanoparticles per Rh6G molecule.

**Figure 2:**
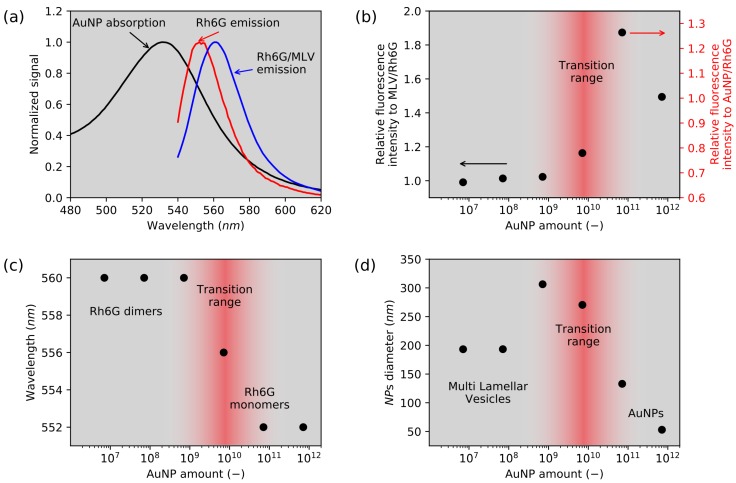
(**a**) normalized Au nanoparticles absorption spectrum (black); rhodamine 6G emission spectra in water solution (red), and MLV (blue); (**b**) relative fluorescence intensity of samples containing Rh6G, MLV with AuNPs measured according to the liposomes suspension with Rh6G; (**c**) variance of maximum Rh6G fluorescence wavelength dependence as a function of the AuNP concentration in the presence of α-PC lipids; (**d**) mean hydrodynamic diameter of samples containing MLV, Rhodamine 6G and AuNPs in a function of nanoparticle amount. At the lowest concentration, only MLVs are visible, but, at higher concentrations, only AuNP are detected by the DLS (*d* = 53 nm).

The fluorescence emission have been observed on the quasi-2D MLV samples in a function of AuNP amount. Normalized spectra of AuNP absorption and Rh6G fluorescence emission with and without liposomes were presented in Figure 2a. The Rh6G solution presented maximum fluorescence emission at $\lambda_{mon}=552$ nm corresponding to the Rh6G monomers emission. In the MLV system, the emission is shifted to $\lambda_{J}=560$ nm. Such behavior is related to the J-aggregates formation [44,45]. This phenomenon (already presented in a previous study [46]) has its origin in the lipophilic and cationic nature of Rh6G molecules interacting with amphiphilic lipid bilayers [47]. With an amount of AuNPs lower than $4.8\times 10^{9}$, a similar emission wavelength $\lambda_{J}=560$ nm (Figure 2c) and a fluorescence intensity (Figure 2b) comparable to that of MLV/Rh6G sample are observed. For this low concentration range, AuNPs have no impact on the MLV system. This was confirmed with DLS measurements and a stable hydrodynamic diameter of about 200 nm related to MLV size (Figure 2d).

On the contrary, for the highest AuNP concentration, the system behaves like a liposomes-free system with a fluorescence intensity identical to that of Rh6G/AuNPs sample (Figure 2b), an emission at λ=552 nm from monomers (Figure 2c) and with a hydrodynamic diameter about 53 nm corresponding to AuNPs size (Figure 2d). Clearly, in this case, liposomes are disrupted due to the large amount of CTAC surfactant brought with AuNPs.

Interestingly, a transition regime is observed with intermediate AuNP concentrations. A maximum fluorescence emission enhancement (+20%) is measured for the nanoparticles amount equal to $4.8\times 10^{10}$ as presented in Figure 2b. It indicates that there is a point where emission enhancement is possible in a multi-phase system, for which population of Rh6G aggregates and monomers is stabilized. Due to the plasmonic interactions, the fluorescence enhancement can only be acquired in AuNPs and dye proximity, whereas Rh6G dimerization should present lower fluorescence intensity. Such an effect was obtained with application of MLVs. Making the comparison to the 3D system of AuNPs and Rh6G, the probability that dye molecule is located near a nanoparticle is low; however, the appliance of MLVs forces a dye position at the lipid bilayer interface. The AuNPs stabilized with CTAC, which has structural analogy to α-PC, provide the dragging force necessary to place nanoparticles and dye in the proximity by a lipid bilayer. In addition, this has been presented by the hydrodynamic diameter increase for a specific AuNP amount. The enhancement effect is possible only when the substantial number of dye molecules are placed near a nanoparticle. In the studied system, there is still some distribution of free dye and nanoparticle suspensions. The key effect is related to the confinement and dye aggregation, thus the enhancement is observed with the utilization of physicochemical oriented assembly and not as the simple dispersion of the dye with the gold nanoparticle.

### 3.2. Lasing Enhancement in PVA: 2D System

**Figure 3:**
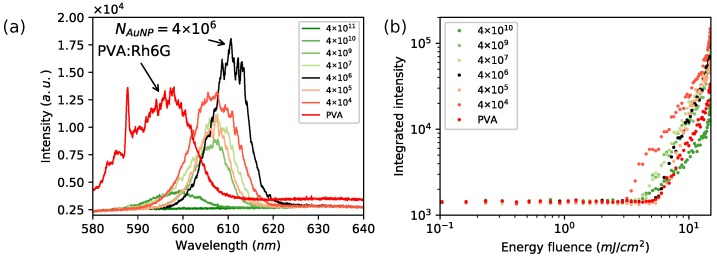
(**a**) random lasing spectra for PVA doped AuNPs and Rh6G. Lasing enhancement was observed for the total number of nanoparticles in the PVA layer equal to $4.0\times 10^{6}$ particles; (**b**) integrated intensities as a function of energy density for lasing threshold determination.

**Figure 4:**
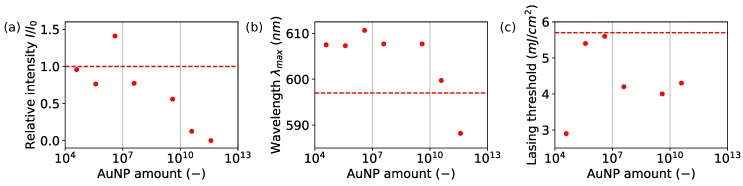
(**a**) relative maximum intensity of random lasing; (**b**) maximum wavelength of lasing emission and (**c**) change in lasing threshold as a function of AuNP amount in the dye doped PVA matrix.

Solid thin layers were subjected to the light amplification experiments in order to visualize AuNPs’ influence effects. Series of samples excited with the nanosecond pulsed laser at wavelength $\lambda_{ex}=532$ nm were induced to obtain population inversion and random lasing action. There were lasing spectra characteristics observed for solid thin films doped with fluorescent dye [48,49]. The position of modes and intensity was changing during excitation, indicating observation of random lasing. Collected lasing emission spectra are presented in Figure 3a, where a strong impact of lasing according to the AuNP amount has been observed. There was acquired nonlinear response vs. increasing AuNP amount, from the reference level measured for Rh6G/PVA, through enhanced lasing up to the emission decay as presented in Figure 4a. For the highest particle number equal to $N_{AuNP}=4.0\times 10^{11},$ the total suppression of lasing occurred as an effect of AuNP absorption at plasmon resonance wavelength. The lasing emission enhancement was observed for the AuNPs at $N_{AuNP}=4.0\times 10^{6}$ of particle number, responsible for the 50% increase in the relative lasing intensity according to the Rh6G/PVA sample (without AuNPs). The excited area of slab waveguide geometry was covered by $N_{AuNP}=2.6\times 10^{4}$ nanoparticles with assumption of uniform distribution. The same parameter estimated for Rh6G indicates that there was $N_{Rh6G}/N_{AuNP}=1.7\times 10^{16}$ Rhodamine molecules for one gold nanoparticle. The volume of a single Au nanoparticle is $V_{AuNP}=2140$ nm${}^{3}$, when a single Rh6G molecule volume is $V_{Rh6G}=0.4$ nm${}^{3}$ according to Penzkofer [50]. Covering the AuNP surface requires *N* = 2378 Rhodamine 6G molecules. The further decreasing nanoparticles amount led to a lowering of emission intensity compared to the reference level accepted for Rh6G:PVA without nanoparticles. The acquired signal has a typical random lasing-like shape with randomly occurring lasing modes. The relationship between the lasing emission intensity as a function of AuNP amount was presented in Figure 3a. Spectra were collected for energy density ρ=14.8 mJ/cm${}^{2}$.

The analysis of lasing action as a function of incident beam energy density presents just slight differences in lasing threshold (Figure 3b and Figure 4c). For the sample showing the highest enhancement, the threshold value was designated as equal to ρth = 5.6 mJ/cm${}^{2}$, where the reference sample lasing threshold was equal to ρth = 5.7 mJ/cm${}^{2}$. The other prepared samples were showing lower thresholds reaching the lowest value equal to ρth = 2.9 mJ/cm${}^{2}$, cf. Table 1, Figure 3b and Figure 4c.

Furthermore, a wavelength emission shift related to the AuNP amount was observed. The highest observed lasing enhancement was correlated with larger maximum emission shift to the longer wavelengths. For the obtained highest intensity, emission occurred at 610 nm, indicating that enhancement was due to higher order aggregates (Figure 4b). This fact is especially worth noticing because Rh6G does not present aggregation induced emission (AIE); then, emission from higher order aggregates is enhanced by gold nanoparticles. Emission was shifted to lower wavelength with increased nanoparticles’ numbers, showing that emission from J-aggregates in proximity to 590 nm is more probable than emission from bigger aggregates.

**Figure 5:**
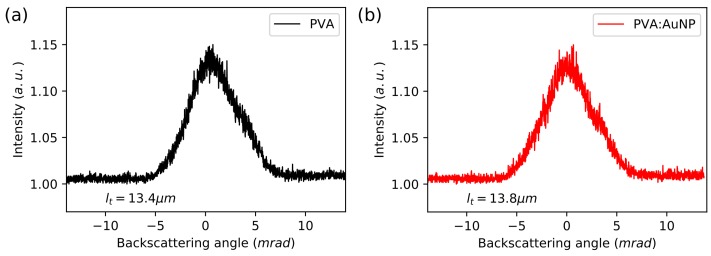
Backscattering cones for PVA doped Rh6G with calculated light transport mean-free path for layer (**a**) without AuNPs and (**b**) with nanoparticles.

The origin of light amplification with the AuNP samples was tested with the CBS experimental technique. The transport mean-free path was calculated using Equation (1). The reference sample of Rh6G/PVA has shown a light transport path equal to $l_{t}=13.4\pm 1.0\;\mu$m, whereas the sample containing $N_{AuNP}=4.0\times 10^{6}$ elevated the value to the $l_{t}=13.8\pm 1.0\;\mu$m. Such a small difference met requirements of device error. Scattering cones are shown in Figure 5. No differences were observed in the light transport mean-free path and the scattering properties, which is coherent with the enhancement mechanism through surface plasmon resonance, and excludes increased scattering or possibility of amplification at a longer light path.

Lowering the dimensionality of a hybrid AuNP system is causing the arrangement of nanoparticles and bringing them in proximity with fluorescent dye causing emission enhancement due to the plasmon resonance. The enhancement occurs for Rh6G monomers as well for aggregates. The observation of dye emission enhancement by nanoparticles mixed with liposomes can be successfully implemented into the nanocompartmentation environments like cells by nanoparticles’ live tracking in dye doped lipid membranes.

## 4. Conclusions

We have demonstrated that hybrid 2D and 3D organizations, obtained by an association of gold nanoparticles with fluorescent molecular dyes without covalent interaction, can provide enhancement of fluorescence emission and random lasing. The property of the three-dimensional system was evaluated in a solution showing a suppressive effect of the emission. Phosphatidylcholine liposomes acted as an intermediate semi-two-dimensional system, where confinement in water suspension forced the proximity between Rh6G and nanoparticles responsible for the fluorescence emission enhancement by 20%. In the two-dimensional system, nanoparticles doped PVA layers were interestingly showing lasing enhancement over 50%. The enhancement originates from the nanoparticles’ plasmon resonance effect, confirmed by the stable value of the light transport mean-free path. The design and fabrication of efficient lasing devices might be performed and enhanced by utilizing plasmon properties of metal nanoparticles concerning only self-organization of complexed structures.

## Figures and Tables

**Table 1:** Random lasing properties from AuNPs, Rh6G doped PVA layers, where Rh6G:PVA ratio is equal to 2.0%(w/w).

Sample	AuNP Amount	Lasing Threshold	Relative Intensity
	*N*	ρth [mJ/cm${}^{2}$]	$I/I_{0}$
*reference*	0.0	5.7	1.00
1	$4.0\times 10^{11}$	*no emission*	0.00
2	$4.0\times 10^{10}$	4.3	0.01
3	$4.0\times 10^{9}$	4.0	0.53
4	$4.0\times 10^{7}$	4.2	0.78
5	$4.0\times 10^{6}$	5.6	1.54
6	$4.0\times 10^{5}$	5.4	0.77
7	$4.0\times 10^{4}$	2.9	0.96
